# Metabolic Alterations and Increased Liver mTOR Expression Precede the Development of Autoimmune Disease in a Murine Model of Lupus Erythematosus

**DOI:** 10.1371/journal.pone.0051118

**Published:** 2012-12-04

**Authors:** Laia Vilà, Núria Roglans, Miguel Baena, Emma Barroso, Marta Alegret, Manuel Merlos, Juan C. Laguna

**Affiliations:** 1 Department of Pharmacology and Therapeutic Chemistry, School of Pharmacy, University of Barcelona, Barcelona, Spain; 2 Institute of Biomedicine, University of Barcelona, Barcelona, Spain; 3 CIBER (Centro de Investigación Biomédica en Red) of Physiopathology of Obesity and Nutrition, Barcelona, Spain; Beth Israel Deaconess Medical Center, Harvard Medical School, United States of America

## Abstract

Although metabolic syndrome (MS) and systemic lupus erythematosus (SLE) are often associated, a common link has not been identified. Using the BWF1 mouse, which develops MS and SLE, we sought a molecular connection to explain the prevalence of these two diseases in the same individuals. We determined SLE- markers (plasma anti-ds-DNA antibodies, splenic regulatory T cells (Tregs) and cytokines, proteinuria and renal histology) and MS-markers (plasma glucose, non-esterified fatty acids, triglycerides, insulin and leptin, liver triglycerides, visceral adipose tissue, liver and adipose tissue expression of 86 insulin signaling-related genes) in 8-, 16-, 24-, and 36-week old BWF1 and control New-Zealand-White female mice. Up to week 16, BWF1 mice showed MS-markers (hyperleptinemia, hyperinsulinemia, fatty liver and visceral adipose tissue) that disappeared at week 36, when plasma anti-dsDNA antibodies, lupus nephritis and a pro-autoimmune cytokine profile were detected. BWF1 mice had hyperleptinemia and high splenic Tregs till week 16, thereby pointing to leptin resistance, as confirmed by the lack of increased liver P-Tyr-STAT-3. Hyperinsulinemia was associated with a down-regulation of insulin related-genes only in adipose tissue, whereas expression of liver mammalian target of rapamicyn (mTOR) was increased. Although leptin resistance presented early in BWF1 mice can slow-down the progression of autoimmunity, our results suggest that sustained insulin stimulation of organs, such as liver and probably kidneys, facilitates the over-expression and activity of mTOR and the development of SLE.

## Introduction

From the second half of the twentieth century onwards, there has been a growth in the prevalence of two apparently unrelated pathologic conditions, namely metabolic and autoimmune diseases, especially in affluent western societies [1]–[5]. This occurrence has coincided with a drastic change in life style, involving massive adoption of sedentary practices associated with dietary habits skewed towards the consumption of high caloric density, nutrient poor foods, which promote a marked positive energy balance in the general population [6], [7].

These life style changes constitute a risk factor for predisposition to metabolic diseases (obesity, insulin resistance, metabolic syndrome, etc.) and their cardiovascular manifestations, such as angina pectoris and myocardial infarction [6], [8]. In the past 25 years the prevalence of some of the risk factors for cardiovascular disease (i.e. cigarette smoking, dyslipaemia, etc.) has gradually declined; however, the prevalence of obesity, metabolic syndrome and diabetes mellitus has dramatically increased as a result of unhealthy changes in dietary habits and life style [3], [7]. Besides, a clear risk factor (excluding the increased concentration of chemicals in the environment of urban areas) related to the increased prevalence of autoimmune diseases has not been identified to date.

Systemic lupus erythematosus (SLE) is an heterogeneous autoimmune disease that affects multiple organs, and its highest prevalence is reported in Italy, Spain, Martinique and the UK Afro-Caribbean population [5], [7]. The disease progresses through four broad stages, which are in the following order: the presence of autoantibodies against a variety of ubiquitous self-antigens; the deposition of autoantibodies and immune complexes in tissues; the development of tissue inflammation; and tissue damage and fibrosis. There is a clear predominance of SLE in females, with female-to-male ratios between 9∶1 and 13∶1. There has been a marked increase in five-year survival from less than 50% in the 1950s to more than 90% in the 1990s as a result of improved therapeutics. Those who die early in the course of SLE have active disease and a high incidence of infection associated with treatment with large doses of corticosteroids, while most patients who die later in the course of the disease, the most common situation nowadays, die from myocardial infarction [9], .

Patients with SLE are five to six times more likely to have a significant coronary event than the general population [9]. Epidemiological studies point to an association of metabolic syndrome (MS) and SLE, indicating that traditional risk factors for cardiovascular disease clustered in the MS, such as hypertension, insulin resistance, hepatic steatosis, diabetes mellitus and obesity, have a significant role in the development of premature atherosclerosis in patients with SLE [11], [12]. There is growing epidemiological evidence that obesity increases the risk of autoimmnune diseases [13], but it is unknown whether these pathological conditions share common molecular pathways.

**Figure 1:**
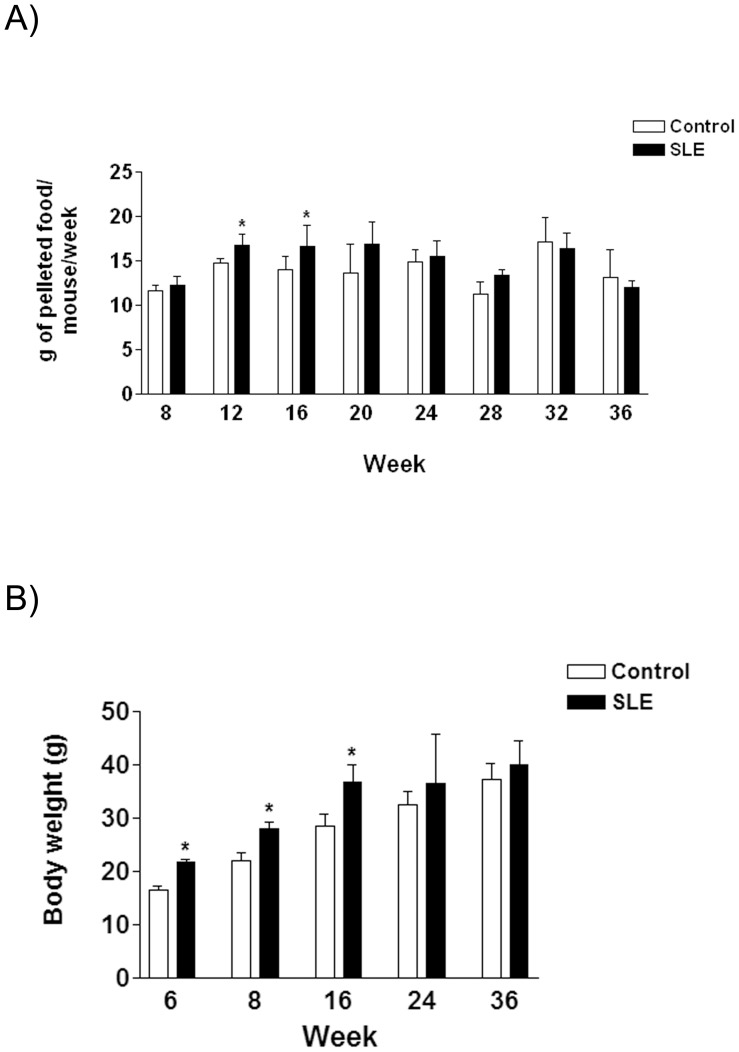
A. Bar diagram showing solid food consumption, expressed as the mean±sd of g of pelleted diet consumed per day and animal, at different times (weeks) for control and SLE mice (8 animals per group). **B.** Body weight, expressed as the mean±sd in g at the beginning of the study (week 6) and for control and SLE mice (8 animals per group) at the weeks of sacrifice. * *P*<0.05.

The New Zealand Black (NZB) mouse is characterized by mild SLE-like symptoms. F1 progeny from NZB mice and the non-autoimmune New Zealand White (NZW) strain, called BWF1, exhibit an earlier onset and a high incidence of SLE manifestations, showing many features of human SLE, including a complex genetic origin, a bias for the female sex, immune complex glomerulonephritis, and the presence of antinuclear antibodies [14]. In a seminal article published by Ryan et al. [15], it was shown that, like humans, the BWF1 mouse model presents several characteristics of the MS, including hypertension, central obesity, insulin resistance, hepatic steatosis and hyperleptinemia.

Here we studied the temporal evolution of SLE and MS manifestations in the murine BWF1 model, with the aim to identify possible molecular connections that could help to explain the high prevalence of these two diseases in the same individuals. We show that MS symptoms appear before those of SLE and propose a possible interconnection of both diseases through hyperinsulinemia and mammalian target of rapamicyn (mTOR) up-regulation.

**Figure 2:**
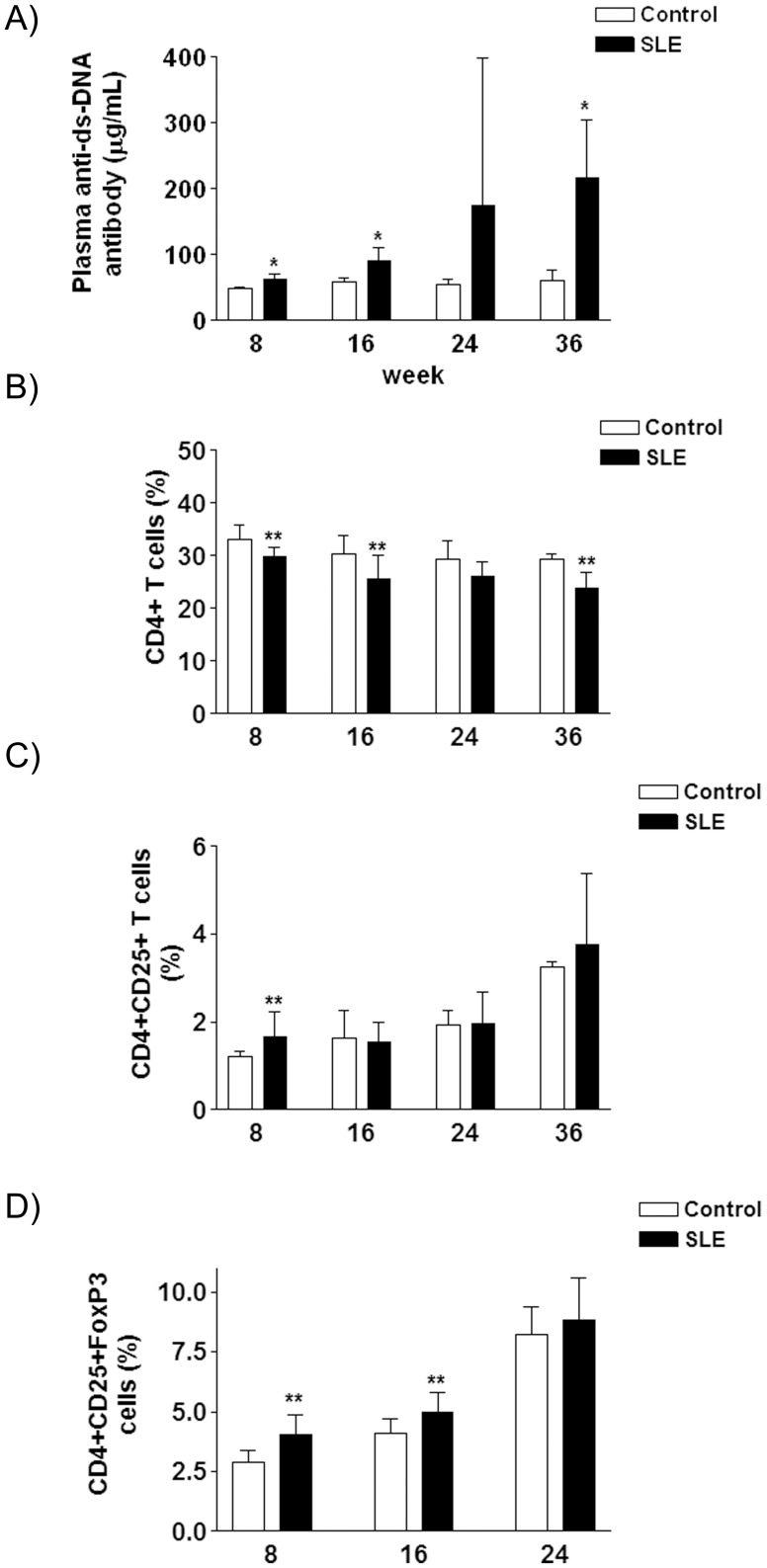
A. Bar diagram showing plasma anti-ds-DNA antibody concentrations expressed as the mean±sd of 8 animals, for control and SLE mice (8 animals per group) at the weeks of sacrifice. B–D. Percentages of CD4^+^- (**B**), CD4^+^CD25^+^- (**C**), and CD4^+^CD25^+^FoxP3-T cells (**D**) in spleen lymphocytes, expressed as the mean±sd of 8 animals, for control and SLE mice (8 animals per group) at the weeks of sacrifice. * *P*<0.05, ** *P*<0.01.

**Figure 3:**
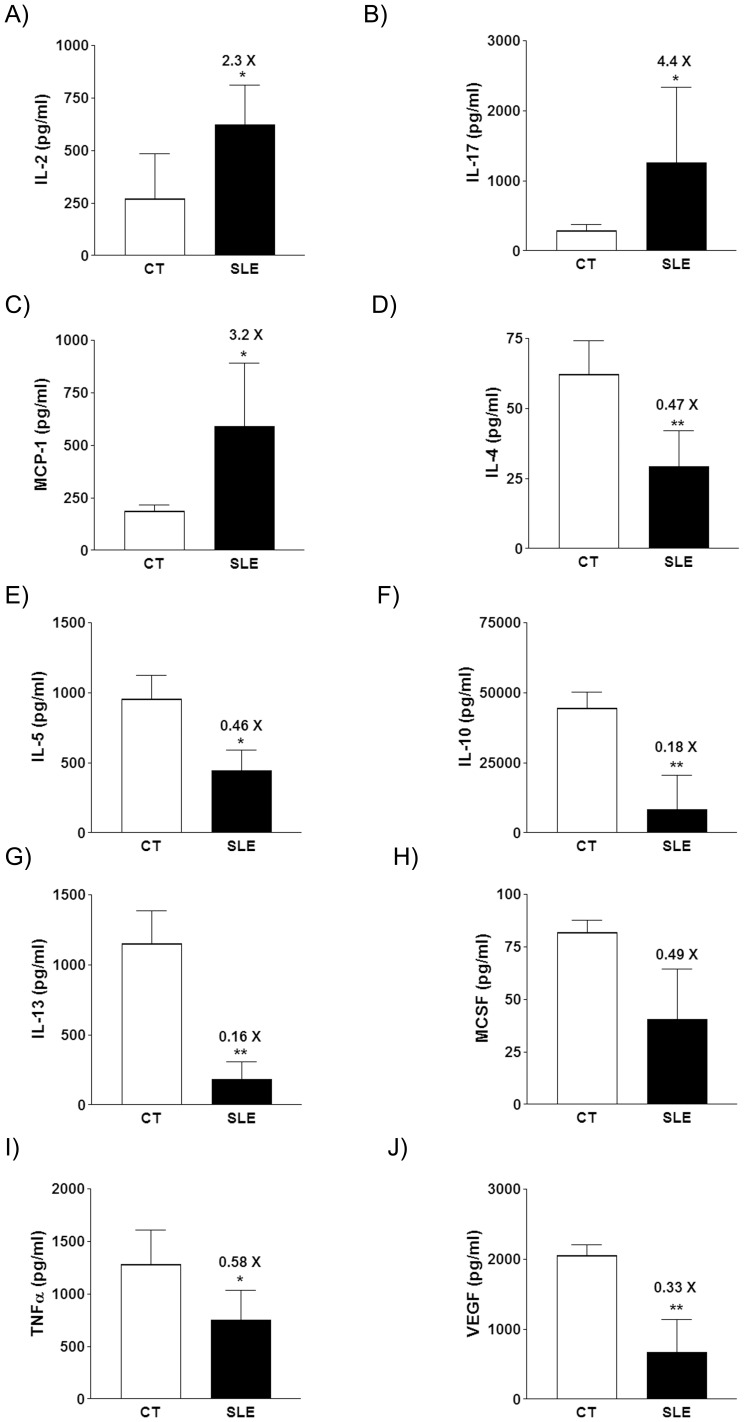
Bar diagrams showing IL-2 (A), IL-17 (B), MCP-1 (C), IL-4 (D), IL-5 (E), IL-10 (F), IL-13 (G), M-CSF (H), TNFα (I), and VEGF (J) concentrations in the supernatant of stimulated cultured splenocytes (see Material and Methods), expressed as the mean±sd of 8 cultures from separate animals, for control and SLE mice at week 36. * *P*<0.05, ** *P*<0.01.

**Figure 4:**
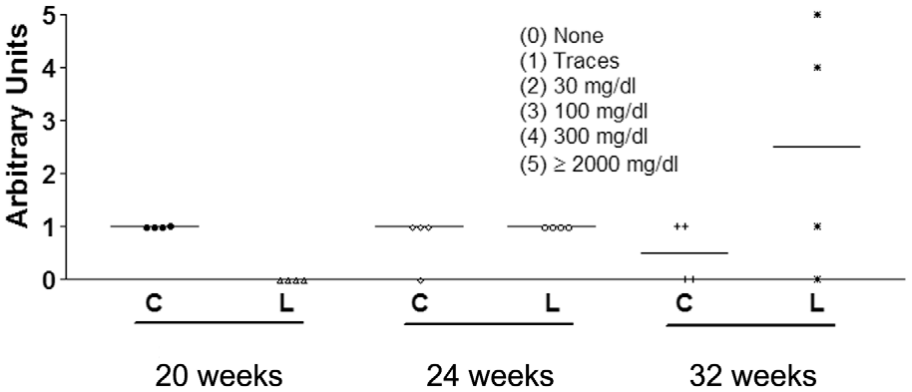
Plot of individual 24 h proteinuria values, expressed as arbitrary units, for control (C) and BWF1 mice (L) at weeks 20, 24 and 32.

**Figure 5:**
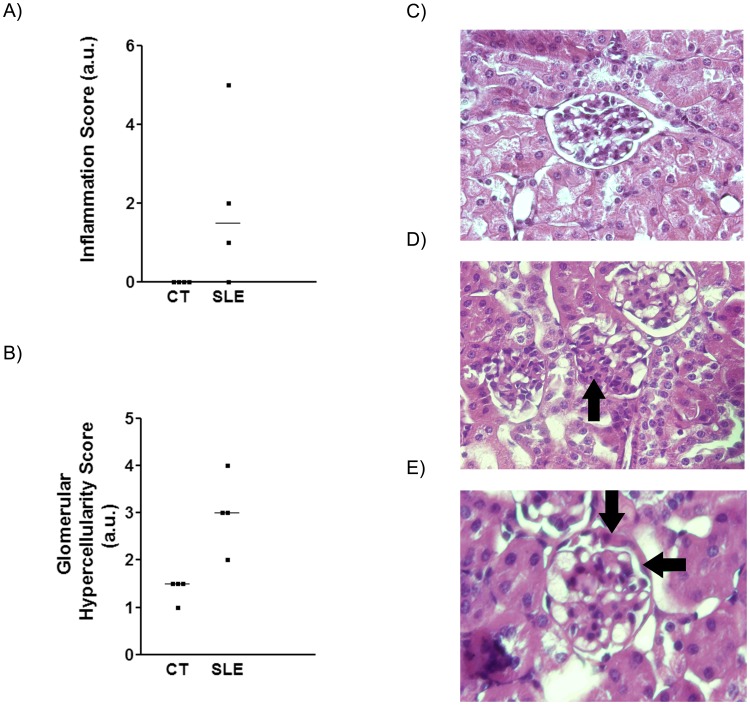
BFWF1 mice, aged 36 weeks, showed increased kidney pathology. Hystological analysis of individual inflammatory (**A**) and glomerular hypercellularity scores (**B**) for CT and SLE mice, evaluated as described in the Material and Methods section. Representative photographs of renal sections from control (CT) (**C**) and BWF1 (SLE) (**D,E**) mice at the age of 36 weeks (H&E staining, light microscopy, original magnification ×40). Arrows indicate mesangial glomerular proliferation (**D**) and cellular extracapillary half-moon (**E**).

## Materials and Methods

### Animals and Experimental Design

Thirty-two female F1 progeny from New Zealand Black/White mice characterized by SLE-like symptoms (SLE) and 32 New Zealand White female mice used as a control (CT) were provided by Charles River Laboratories (Barcelona, Spain). All mice were maintained with access to water and food *ad libitum* and under constant humidity and temperature with a light/dark cycle of 12 hours. After 8, 16, 24 and 36 weeks the mice were killed under isoflurane anesthesia between 9–10 a.m. During the study, we periodically measured solid food consumption and the body weight.

**Figure 6:**
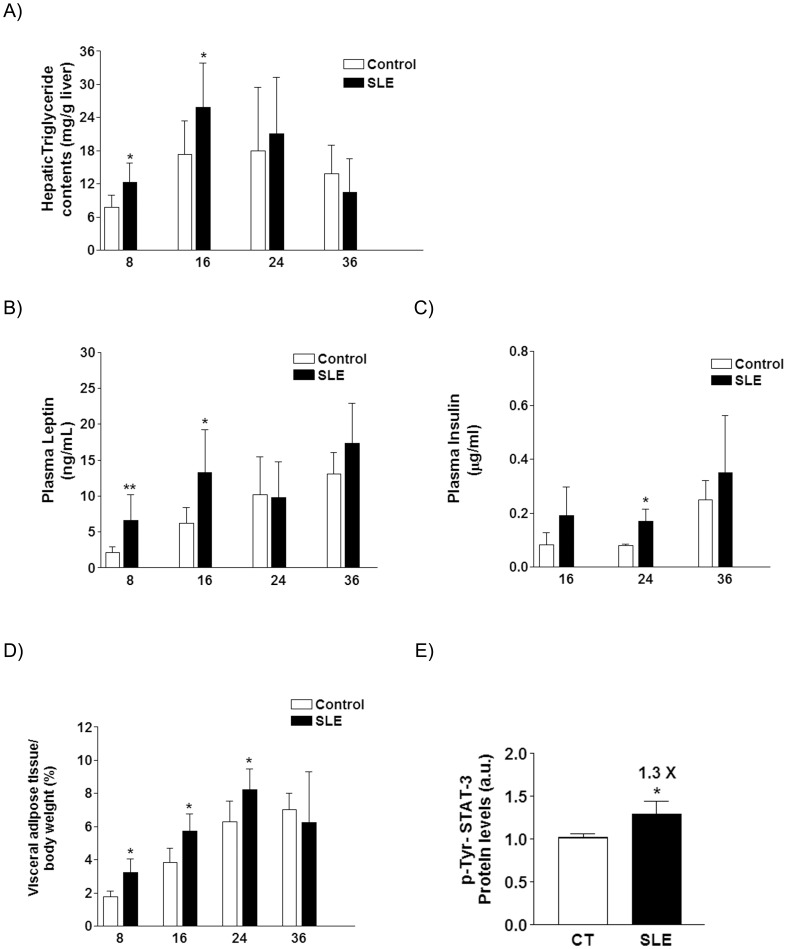
Bar diagrams showing hepatic triglyceride content (A), plasma leptin (B), and insulin concentrations (C), and percentage of adipose visceral tissue (D), expressed as the mean±sd of 8 animals, for control and SLE mice at the weeks of sacrifice. **E.** Bar diagrams showing the relative amount of P-Tyr-STAT-3 protein in liver tissue, expressed as the mean ± sd of values from 8 animals, from control (CT) and BWF1 (SLE) mice. The amount of protein loaded was confirmed by the Bradford method, and the uniformity of protein loading in each lane was assessed by determining the signal of β–actin as a control-loading protein.* *P*<0.05, ** *P*<0.01.

**Figure 7:**
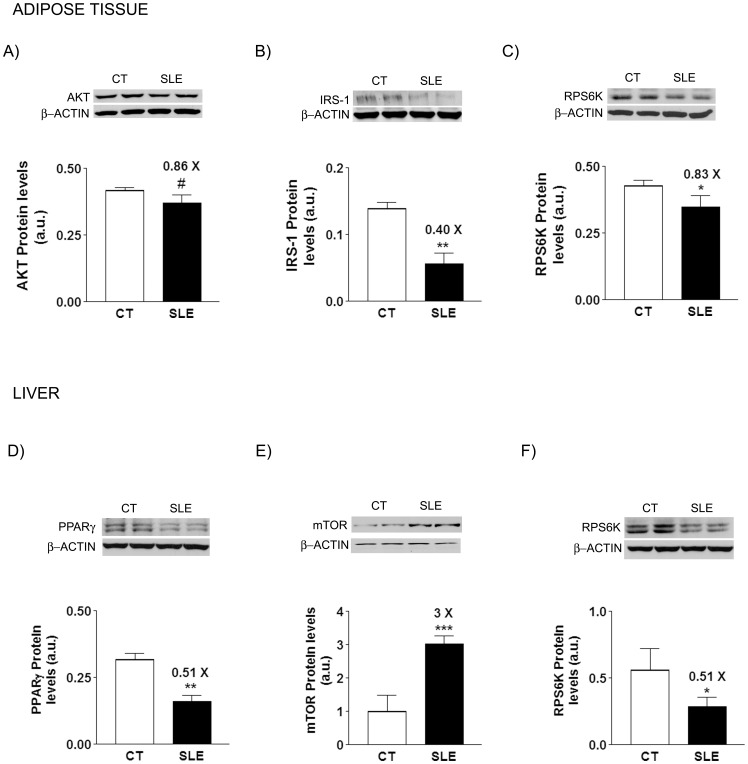
Bar diagrams showing the levels of AKT (A), IRS-1 (B) and RPS6K (C) protein in adipose tissue, and PPARγ (D), mTOR (E), and RPS6K (F) protein in liver tissue, expressed as the mean ± sd of values from 8 animals aged 16 weeks, for control (CT) and BWF1 (SLE) mice. The amount of protein loaded was confirmed by the Bradford method, and the uniformity of protein loading in each lane was assessed by determining the signal of β–actin as a control-loading protein. A representative autoradiography from a Western blot determination of two animals per group is shown. # *P* = 0.05, * *P*<0.05, ** *P*<0.01, *** *P*<0.001.

**Table 1:** Genes whose expression was altered in livers from 16-week old SLE *vs* CT mice.

Gene	Name	Fold change	95% CI	P
*Dok1*	Docking protein 1	0.64	0.46–0.82	0.030
*Dusp14*	Dual specificity phosphatase 14	0.82	0.74–0.89	0.017
*Eif2b1*	Eukaryotic translation initiationfactor 2B, subunit 1 (alpha)	0.23	0.00–0.67	0.019
*Eif4ebp1*	Eukaryotic translation initiationfactor 4E binding protein 1	0.02	0.00–0.11	0.010
*Frap1*	FK506 binding protein 12-rapamycin associated protein 1	0.56	0.42–0.70	0.005
*Gpd1*	Glycerol-3-phosphatedehydrogenase 1 (soluble)	0.70	0.49–0.90	0.044
*Hras1*	Harvey rat sarcoma virusoncogene 1	4.78	3.80–5.77	0.000
*Lep*	Leptin	0.38	0.19–0.58	0.014
*Pck2*	Phosphoenolpyruvatecarboxykinase 2 (mitochondrial)	0.54	0.36–0.72	0.022
*PPARg*	Peroxisome proliferatoractivated receptor gamma	0.57	0.46–0.68	0.002
*Raf1*	V-raf-leukemia viral oncogene 1	0.61	0.43–0.80	0.015
*Rps6ka1*	Ribosomal protein S6 kinasepolypeptide 1	0.65	0.50–0.81	0.015
*Klf10*	Kruppel-like factor 10	0.43	0.25–0.61	0.015

**Table 2:** Genes whose expression was altered in visceral adipose tissue from 16-week old SLE *vs* CT mice.

Gene	Name	Fold change	95% CI	P
*Acaca*	Acetyl-Coenzyme A carboxylase alpha	0.07	0.00–0.27	0.029
*Aebp1*	AE binding protein 1	0.01	0.00–0.03	0.036
*Akt1*	Thymoma viral proto-oncogene 1	0.07	0.00–0.20	0.026
*Akt3*	Thymoma viral proto-oncogene 3	0.11	0.00–0.43	0.027
*Araf*	V-raf murine sarcoma 3611 viral oncogenehomolog	0.18	0.00–0.46	0.046
*Bcl2l1*	Bcl2-like 1	0.10	0.00–0.37	0.010
*Fbp1*	Fructose bisphosphatase 1	0.47	0.24–0.70	0.027
*Frs3*	Fibroblast growth factor receptor substrate 3	0.10	0.00–0.35	0.034
*Gab1*	Growth factor receptor bound protein2-associated protein 1	0.11	0.00–0.34	0.031
*Grb10*	Growth factor receptor bound protein 10	1.70	1.44–1.96	0.003
*Igf1r*	Insulin-like growth factor I receptor	0.05	0.02–0.09	0.010
*Irs1*	Insulin receptor substrate 1	0.33	0.10–0.57	0.028
*Jun*	Jun oncogene	0.07	0.00–0.17	0.028
*Mapk1*	Mitogen-activated protein kinase 1	0.29	0.04–0.54	0.048
*Nck1*	Non-catalytic region of tyrosine kinase adaptor protein 1	0.43	0.09–0.76	0.050
*Pck2*	Phosphoenolpyruvate carboxykinase 2 (mitochondrial)	0.16	0.00–0.32	0.004
*Pik3r2*	Phosphatidylinositol 3-kinase, regulatory subunit, polypeptide 2 (p85 beta)	0.07	0.00–0.24	0.023
*Ppp1ca*	Protein phosphatase 1, catalytic subunit, alpha isoform	0.08	0.00–0.26	0.015
*Prkcz*	Protein kinase C, zeta	0.26	0.00–0.55	0.004
*Ptpn1*	Protein tyrosine phosphatase, non-receptor type 1	0.04	0.00–0.13	0.001
*Retn*	Resistin	0.20	0.00–0.52	0.023
*Rps6ka1*	Ribosomal protein S6 kinase polypeptide 1	0.07	0.00–0.21	0.011
*Slc27a4*	Solute carrier family 27 (fatty acid transporter),member 4	0.09	0.00–0.27	0.045
*Slc2a1*	Solute carrier family 2 (facilitated glucosetransporter), member 1	0.05	0.00–0.13	0.008

For each group, four mice were transferred to metabolic cages and kept 24 h for urine collection. Urinary protein excretion (proteinuria) was determined by Albustix^R^ (Bayer, Barcelona, Spain) once every 4 weeks starting from 8 week of the study, always using the same animals.

When the animals were killed, blood was collected in 5% EDTA-tubes for measurement of serum anti-double stranded DNA (anti-dsDNA) antibody levels, lipids, glucose, creatinine, insulin and leptin concentrations. Kidneys were harvested for histological studies; spleens were also harvested and washed with PBS-FBS 10% (10% heat inactivated fetal bovine serum) and 1% antibiotic solution for isolation and culture of splenocytes. Hepatic and adipose tissues were excised, weighed and immediately frozen in liquid N_2_.

All procedures were conducted in accordance with the guidelines established by the University of Barcelona’s Bioethics Committee, as stated in Law 5/1995 (21st July) drawn up by the Generalitat de Catalunya.

### Plasma Analysis

Blood samples were centrifuged to obtain plasma and then stored at −20°C until needed. Plasma triglyceride, glucose concentrations and creatinine levels were measured using the colorimetric tests from SPINREACT (Girona, Spain) (ref. 1001312, 1001192 and K-4001, respectively). Non-esterified fatty acids (NEFA) were measured using a colorimetric test from Wako Chemicals GmbH (Neuss, West Germany). Plasma insulin and leptin concentrations were determined with the Insulin RIA kit (RI-13K) and Leptin RIA kit (RL-83K) from Linco Research (Missouri, USA), respectively.

Plasma anti-dsDNA antibody concentration was measured by an ELISA kit from Alpha Diagnostic International (Texas, USA), following the manufacturer’s instructions.

### Hepatic Triglyceride Contents

Liver triglyceride levels were measured as described previously [16] and were determined using the same colorimetric test from SPINREACT (Girona, Spain) as described above.

### Fatty Acid Oxidation Activity

Hepatic fatty acid β-oxidation activity was determined as described previously [17], using 30 µg of post nuclear supernatant from each sample.

### Western Blot Analysis

150 mg of hepatic and adipose tissue from each animal were homogenized in a buffer containing 150 mM NaCl, 1 mM EDTA, 1 mM EGTA, 1% Igepal, 100 mM NaF, 1 mM each of PMSF, Nappi, sodium ortovanadate and 20 mM Tris-HCl pH 7.5 buffer to obtain the total protein fraction by centrifugation. This fraction was stored at −80°C until needed. The protein concentration was determined by the Bradford method [18].

30 µg of total protein (15 µg for IRS-1 experiments) were subjected to 10% SDS-polyacrylamide gel electrophoresis. Proteins were then transferred to Immobilon polyvinylidene diflouride transfer membranes (Millipore, Bedford, MA) and blocked for 1 h at room temperature with 5% non-fat milk solution in TBS-0.1% Tween-20. Membranes were then incubated with the primary polyclonal antibody raised against total-AKT, IRS-1, and RPS6K (dilution 1∶1000) in adipose tissue samples and PPARγ, mTOR, RPS6K and p-Tyr-STAT-3 (dilution 1∶1000) in liver samples in TBS-0.1% Tween-20 with 5% non-fat milk at 4°C overnight. After several washes, the membranes were incubated with horseradish peroxidase-conjugated anti-rabbit IgG (1∶3000 dilutions). Detection was achieved using the ECL chemiluminescence kit for HRP (GE Healthcare Bio-Sciences AB, Uppsala, Sweden). To confirm the uniformity of protein loading in each lane, the blots were incubated with β-actin protein. The size of the proteins detected was estimated using protein molecular-mass standards (BioRad Laboratories SA, Barcelona, Spain). All antibodies were obtained from Cell Signaling Technology Inc. (Danvers, USA).

### PCR- Arrays

Six total RNA pools from liver and six total RNA pools from adipose tissue were prepared, three for CT mice and three for SLE counterparts. Each pool was prepared with the same amount of RNA from two mice, with a total of 6 mice in each condition. Briefly, total RNA was isolated using TRIzol reagent (Invitrogen-Life Technologies, New York, USA) following the manufacturer’s instructions and was then purified using RNeasy kit columns (Qiagen Iberia S.L., Madrid, Spain). Single stranded cDNA and PCR arrays were performed using the RT^2^ Profiler™ PCR Array Mouse Insulin Signaling Pathway (PAMM-030A) from SaBiosciences (Madison, USA) and following the manufacturer’s guidelines. Array data processing and analysis were performed by using a Web portal from SaBiosciences (Madison, USA).

### Renal Histology

Kidney specimens were fixed in 10% formaldehyde and embedded in paraffin. Four µm sections were stained with haematoxylin and eosin (H&E). Inflammatory cell infiltration and glomerular hypercellularity were evaluated semi-quantitatively by a renal pathologist blinded to group assignment (arbitrary score 0 to 3+, where 0 = no change; 1+ = mid; 2+ = moderate and 3+ = severe).

### Isolation of Splenocytes

The isolated spleen from each mouse was pressed through a sterile 40 µm nylon cell strainer (Sigma-Aldrich, St. Louis, CA, USA) to make a single cell suspension. After centrifugation at 538×g for 10 min at 4°C, red blood cells in the pellet were lysed by osmotic shock with PBS 1X and distilled water. In order to restore tonicity, PBS 10X was added and the solution was vortexed and centrifuged at 538×g for 10 min at 4°C. The pellet was then resuspended with PBS-FBS10%. The cells were then counted and viability was determined by the trypan blue exclusion method.

### Labeling of Isolated Splenocytes and Flow Cytometry

5×10^5^ isolated spleen cells were distributed into FACS tubes with 1 ml of PBS-FBS 2% and cold 1% NaN_3_. After centrifugation at 538×g for 5 min at 4°C, the pelleted cells were resuspended and stained with 10 µl of anti-mouse antibodies conjugated by fluorescent dyes (mouse anti-CD4 antibody PE conjugated, mouse anti CD25 FITC conjugated and human anti-CD7 antibody used as a control (eBiosciences, St. Diego, CA, USA). They were then incubated at 4°C for 20 min. After one wash with cold PBS, stained cells were centrifuged and permeabilized using 1X Permeabilizing Solution (ref. 00–5521, eBiosciences, St. Diego, CA, USA) and then incubated for 30 min at 4°C with 15 µl of anti-mouse/rat foxp3 antibody conjugated with APC. Cells were fixed using 500 µl of 0.5% paraformaldehyde solution (p-formaldehyde 2.5 g and NaCl 4.2 g) and 10.000 cells were analyzed by flow cytometry. Results were expressed as the percentage of surface CD4 and CD25 markers and foxp3 in total lymphocytes.

### Measurement of Cytokine Production

The resuspended splenocytes (2×10^6^ cells/ml) were stimulated in 2.5 µg/ml anti CD3 mAb-coated plates and incubated for three days at 37°C under 5% CO_2_ and 95% air. Cells were harvested from 6-well culture plates and the supernatant medium was stored at −20°C. The amounts of cytokines in splenocytes culture media were measured by commercial semi-quantitative cytokine antibody array (Quantibody^R^ Mouse Cytokine Array I, QAM-CYT-1, from Ray Biotech, Inc., Atlanta, GA, USA) following the manufacture’s protocol. 100 µl of sample was used for each condition.

### Statistical Analysis

The results are expressed as the mean of *n* values ± standard deviation. Plasma samples were assayed in duplicate. Significant differences were established by the unpaired *t*-test, using the computer program GraphPad InStat (GraphPad Software V2.03). When the variance was not homogeneous, a non-parametric test was performed (Mann-Whitney). The level of statistical significance was set at *P*<0.05.

## Results

### Food Consumption and Body Weight Evolution

CT and BWF1 (SLE) mice showed a healthy appearance throughout the study. No death from unexpected causes or due to ethical euthanasia was recorded in either group. From the beginning of the study up to week 16, SLE mice consumed a higher amount (x1.18 fold, Figure 1A) of solid food than CT mice. Accordingly, during the same time period SLE mice showed a higher body weight (x1.18 fold, Figure 1B) than CT animals.

### Markers of Systemic Lupus Erythematosus

The plasma anti-ds-DNA antibody concentration rose steadily in SLE mice in comparison with CT animals, from a ×1.3 fold increase at week 8 to a ×3.5 fold increase at week 36 (Figure 2A). SLE mice consistently showed a modest reduction (between ×0.81–x0.90 fold *vs* CT values) in the percentage of CD4+ T cells in spleen lymphocytes (Figure 2B). Despite this reduction, the percentage of CD4+CD25+ T cells was increased at week 8 (x1.4 fold *vs* CT values, Figure 2C), and the percentage of CD4+CD25+FoxP3 T cells increased in SLE mice at week 8 (x1.4 fold *vs* CT values) and 16 (x1.16 fold *vs* CT values) (Figure 2D).

Cytokine production by splenocytes was measured at week 8, 16, 24 and 36. The concentration of almost every cytokine in the supernatant of splenocytes from both SLE and CT mice increased throughout the study, except for granulocyte macrophage-colony stimulating factor (GM-CSF), interleukin (IL) -3, IL-12, IL-17, keratinocyte-derived cytokine (KC), and monocyte chemotactic protein-1 (MCP-1) (data not shown). At week 36, the concentrations of IL-2 (x2.3 fold, Figure 3A), IL-17 (x4.4 fold, Figure 3B), and MCP-1 (x3.2 fold, Figure 3C) were higher in samples from SLE mice than CT ones, while the concentrations of IL-4 (x0.47 fold, Figure 3D), IL-5 (x0.46 fold, Figure 3E), IL-10 (x0.18 fold, Figure 3F), IL-13 (x0.16 fold, Figure 3G), monocyte/macrophages colony stimulating factor (M-CSF) (x0.49 fold, Figure 3H), tumor necrosis factor α (TNFα) (x0.58 fold, Figure 3I), and vascular endothelial growth factor (VEGF) (x0.33 fold, Figure 3J) were lower in samples from SLE mice.

Renal function abruptly worsened in SLE mice by the end of the study. While plasma creatinine concentrations did not vary along the study (data not shown), 24 h proteinuria was higher in SLE than in CT mice by week 32 (Figure 4). In agreement, analysis of H&E-stained kidney sections obtained at week 36 showed a higher inflammation and glomerular hypercellularity score in tissue samples from SLE than from CT mice (Figure 5).

### Markers of Metabolic Syndrome

SLE mice showed hepatic steatosis at weeks 8 and 16 (x1.5 fold *vs* CT for liver triglyceride content), which disappeared at later time points (Figure 6A), with no change in hepatic fatty acid β-oxidation activity (data not shown). Of the several plasma analytes measured (triglycerides, NEFA, glucose, leptin and insulin) only leptin and insulin concentrations were changed in SLE vs CT mice (Figure 6B and 6C). SLE mice were hyperleptinemic at weeks 8 and 16 (x3.1 and ×2.1 fold *vs* CT, respectively), and hyperinsulinemic throughout the entire study (x2.2 fold *vs* CT, at week 16 the difference was not significant). Furthermore, the ratio between visceral adipose tissue and body weight was higher in SLE than in CT mice, although the difference progressively decreased throughout the study, from an increase of ×1.83 fold at week 8, to a ×1.17 fold at week 24, with no significant difference at the end of the study (week 36) (Figure 6D). Despite the hyperleptinemia, livers of 16-week-old SLE animals had no increased levels of P-Tyr-STAT3 protein (Figure 6E), a marker of leptin activity. As maximal differences in the MS between SLE and CT mice clustered around week 16, at this time point we used a commercial PCR Array to characterize the expression of 85 genes involved in insulin signaling in liver and visceral adipose tissue samples. In the liver, 12 genes were down-regulated and 1 gene was up-regulated in SLE *vs* CT mice, while in visceral adipose tissue, 23 genes were down-regulated and 1 gene was up-regulated in SLE *vs* CT mice (Tables 1 and 2). Of all these genes, only *pck2* (phosphoenolpiruvate carboxykinase 2) and *rps6k* (ribosomal protein serine 6 kinase) were similarly down-regulated in both tissues. We selected some key genes in order to elucidate whether changes in mRNA were translated into similar alterations in protein levels. Thus, we determined the amount of total Akt (thymoma viral proto oncogen), IRS-1 (insulin receptor substrate-1), and RPS6K protein in visceral adipose tissue samples, as well as PPARγ(peroxisome proliferator activated receptor γ), FRAP1 (FK506 binding protein 12-rapamycin associated protein 1 or mTOR –mammalian target of rapamycin), and also RPS6K protein in liver samples. The expression of all these proteins was markedly reduced in samples from SLE *vs* CT (Figure 7), except in the case of mTOR (Figure 7e), which was increased ×3.04 fold in liver samples from SLE *vs* CT, in sharp contrast with the reduction in its specific mRNA levels.

## Discussion

Here we used a murine model of SLE (the BWF1 mouse), which, like humans with this autoimmune disease, shows the classical manifestations of MS. Our results indicate that these metabolic alterations temporally precede the development of lupus symptoms in this model.

Thus, from the beginning of the study until week 16, SLE mice showed clear signs of metabolic alterations that temporally preceded the development of lupus symptoms, such as hyperphagia, hyperleptinemia, hyperinsulinemia, fatty liver and increased visceral adipose tissue. During this period, anti-dsDNA antibody plasma levels were low, and no clear signs of lupus disease were observed. By the end of the study, metabolic disturbances had almost disappeared, except for the increased levels of plasma insulin. Coincident in time with the waning of metabolic alterations, anti-dsDNA antibody reached maximal levels and manifestations of lupus nephritis appeared, exemplified by proteinuria (week 32) and a higher inflammation score and glomerular hypercellularity in renal tissue samples from SLE mice (week 36). Also at week 36, the profile of cytokine production by stimulated splenocytes from these mice was consistent with autoimmunity promotion, with an increased production of type Th1 (IL-2) and Th17 (IL-17) cell cytokines, and fundamentally, a reduced production of type Th2 cell cytokines (IL-4, IL-5, IL-10, and IL-3) [19]–[22]. In a similar study using the same model, Alperovich et al. [23] reported the appearance of lupus-related signs earlier, and mortality was higher than in the present study. Thus, the authors reported a 69% survival rate at week 36, with high plasma anti-dsDNA antibody levels from week 20 and proteinuria from week 28. In our study, survival at week 36 was 100%, with animals showing a marked increase in plasma anti-dsDNA antibody levels at week 24 and proteinuria at week 32. Although we do not know the reason for this discrepancy in the severity of the evolution of lupus manifestations, in our case the metabolic disturbances preceded the appearance of the clinical symptoms of SLE. Once the autoimmune disease flourished, the deep alteration of whole body homeostasis may have obliterated the signs of diseased metabolism, such as the increase in triglyceride accretion in liver or adipose visceral tissue.

The development of autoimmune diseases is promoted by deficiency in a special set of regulatory T cells that are crucial in the maintenance of autologous tolerance and are characterized by the expression of CD4, CD25 and FoxP3 (CD4^+^CD25^+^FoxP3^+^ Tregs) [24]. It has been described that leptin, an adipocytokine derived from body fat stores, reduces the proliferation of Tregs [25]–[27]. However, in our study SLE mice were hyperleptinemic at weeks 8 and 16, while at the same time points they showed increased proportions of natural or splenic Tregs. The observation that Tregs did not decrease suggests a deficit of leptin action on the proliferation of these cells.

Resistance to leptin action in the central nervous system and peripheral organs, such as the liver, has been reported in rodent experimental models and in humans [28], [29]. Although Ryan et al. [15] described that hyperleptinemic BWF1 mice showed no signs of central leptin resistance, their results did not ruled out the possibility of peripheral leptin resistance. To explore this issue, we determined the amount of signal transducer and activator of transcription 3 (STAT3) protein phosphorylated on tyrosine (P-Tyr-STAT3), a marker of leptin receptor activation [30]. We did not detect differences in the amount of P-Tyr-STAT3 in liver samples of SLE or CT animals obtained at week 16 (Figure 6E). This observation confirms leptin resistance in peripheral tissues. It could be hypothesized that young SLE mice are protected from the development of the clinical symptoms of SLE by the increased production of Tregs, which occurs because of peripheral leptin resistance.

As SLE mice also showed mildly elevated plasma insulin concentrations during the entire study, we addressed whether these animals also developed insulin resistance in the liver or visceral adipose tissue. Using a commercial cDNA array specifically designed to determine the expression of genes involved in insulin signaling, we demonstrated a marked reduction in the expression of key genes directly participating in the transmission of this signal in the visceral adipose tissue of SLE mice at week 16. Of the 24 genes modified, 10 genes directly involved in insulin signaling (*Akt1, Akt3, Frs3, Gab1, Igf1r, Irs1, Nck1, Pik3r2, Prkcz,* and *Slc2a1)* were down-regulated. This finding points to insulin resistance in the visceral adipose tissue of SLE mice and suggests that this resistance is responsible for the increase in insulin plasma concentrations detected in these animals. The down-regulation of *Akt,* and *Irs1*, genes coding for two essential proteins in the intracellular transmission pathway of the insulin receptor [31], was confirmed at the protein level.

When we applied the same commercial cDNA array to hepatic samples, the expression of the above mentioned genes was not modified; instead, we detected a marked down-regulation of *Eif2b1, Eif4ebp1,* and *Rps6ka1* in the livers of 16-week-old SLE mice. Proteins coded by these genes are downstream of the signaling pathway of the mTORC1 complex, which is involved in cell growth, translation, and ribosomal protein synthesis [32]. Moreover, the mTORC1 complex is formed by two main components, the proteins Raptor and mTOR [32], the latter coded by *Frap1*, which was also down-regulated in the livers of SLE mice. When we attempted to confirm these results at the protein level, although liver Rps6ka1 protein (S6kinase) was reduced, the amount of mTOR protein was markedly increased. The reason for this discrepancy remains unknown. One can speculate that the mTORC1 system was over-stimulated in the livers of SLE mice. This may elicit a cellular compensatory response in an attempt to decrease the over-activity of the system by down-regulating the mRNA expression of its main components, Eif2b1, Eif4ebp1, and Rps6ka1 and mTOR itself.

Recent data points to an association between the increased prevalence of several types of cancer in type 2 diabetic patients and sustained hyperinsulinemia. In this situation, the continuous activity of insulin in non-resistant tissues promotes the activation of the mTORC1 system, thereby favoring cellular proliferation and tumor development [33]. Furthermore, it has been shown that lupus nephritis in rodent models and humans is directly related to the activation of the mTOR pathway in renal tissue [34], [35]. Also, drugs that inhibit mTOR activity are effective in the treatment of SLE manifestations [4], [5], [23].

As a conclusion, our results indicate that, although metabolic alterations, mainly leptin resistance in the BWF1 mice, slow-down the progression of autoimmunity, the presence of hyperinsulinemia and the sustained insulin stimulation of organs that remain insulin-sensitive, such as the liver and potentially the kidneys, facilitates the overexpression and activity of the mTOR system and the appearance of the clinical symptoms of SLE. As the whole body homeostasis deteriorates, metabolic symptoms, except for increases in plasma insulin concentrations, decline and disappear, while classical SLE symptoms progressively develop. If these findings can be extrapolated to humans, subclinical insulin resistance, sustained over time, could be the key factor for the development of clinical SLE in subjects with an autoimmune-prone genetic background.
